# SPATIAL NAVIGATION SKILLS IN MILD COGNITIVE IMPAIRMENT (MCI) – A META-ANALYSIS

**DOI:** 10.1093/geroni/igad104.3598

**Published:** 2023-12-21

**Authors:** Dorota Kossowska-Kuhn, Neil Charness

**Affiliations:** Florida State University, Tallahassee, Florida, United States; FSU, Tallahassee, Florida, United States

## Abstract

**Background:**

Dementia has a tremendous impact on societies and individuals world-wide. One of the earliest symptoms of Alzheimer’s Disease (AD) is spatial disorientation (Coughlan et al. 2018). Spatial orientation problems commonly manifest in Mild Cognitive Impairment (MCI), which represents a state between cognitively healthy aging and dementia. It appears that spatial navigation skills might be an early indicator of cognitive decline due to MCI.

**Methods:**

The present meta-analysis examines the difference between the performance of cognitively healthy older adults and older adults with MCI on spatial navigation tests. The standardized mean difference (Hedge’s g) is used as the effect size measure. In addition to simple characteristics (year of publication, country of study, age of participants, gender, education), we examined the form of test administration (i.e., real-world, virtual reality), type of measure (time, accuracy) and the additional spatial tasks connected with the main spatial navigation test in moderator analyses.

**Results:**

Current data consists of 69 effect sizes across 26 studies. These studies represent 1359 participants (682 MCI, 677 healthy older adults). The overall result shows that the navigation skills of healthy cognitively older adults are significantly better (p < 0.05) comparing to people with MCI by the standardized mean difference (SMD) = 0.81.

**Conclusion:**

Given the large effect size, it is worthwhile to examine potential moderators. It would also be useful to assess the effect size for spatial navigation in people with Alzheimer’s Disease.

